# Metabolic Equivalent in Adolescents, Active Adults and Pregnant Women

**DOI:** 10.3390/nu8070438

**Published:** 2016-07-20

**Authors:** Katarina Melzer, Juliane Heydenreich, Yves Schutz, Anne Renaud, Bengt Kayser, Urs Mäder

**Affiliations:** 1 Swiss Federal Institute of Sport, Magglingen 2532, Switzerland; Juliane.Heydenreich@baspo.admin.ch (J.H.); Anne.Renaud@baspo.admin.ch (A.R.); Urs.Maeder@baspo.admin.ch (U.M.); 2 Faculty of Biology and Medicine, Department of Physiology, University of Lausanne, Lausanne 1015, Switzerland & Integrative Cardiovascular and Metabolic Physiology, University of Fribourg, Fribourg 1700, Switzerland; Yves.Schutz@unifr.ch; 3 Institute of Sports Science (ISSUL), Faculty of Biology and Medicine, University of Lausanne, Lausanne 1015, Switzerland; Bengt.Kayser@unil.ch

**Keywords:** resting metabolic rate, metabolic equivalent, adolescents, pregnant women, physical activity, active men

## Abstract

“Metabolic Equivalent” (MET) represents a standard amount of oxygen consumed by the body under resting conditions, and is defined as 3.5 mL O_2_/kg × min or ~1 kcal/kg × h. It is used to express the energy cost of physical activity in multiples of MET. However, universal application of the 1-MET standard was questioned in previous studies, because it does not apply well to all individuals. Height, weight and resting metabolic rate (RMR, measured by indirect calorimetry) were measured in adolescent males (*n* = 50) and females (*n* = 50), women during pregnancy (gestation week 35–41, *n* = 46), women 24–53 weeks postpartum (*n* = 27), and active men (*n* = 30), and were compared to values predicted by the 1-MET standard. The RMR of adolescent males (1.28 kcal/kg × h) was significantly higher than that of adolescent females (1.11 kcal/kg × h), with or without the effects of puberty stage and physical activity levels. The RMR of the pregnant and post-pregnant subjects were not significantly different. The RMR of the active normal weight (0.92 kcal/kg × h) and overweight (0.89 kcal/kg × h) adult males were significantly lower than the 1-MET value. It follows that the 1-MET standard is inadequate for use not only in adult men and women, but also in adolescents and physically active men. It is therefore recommended that practitioners estimate RMR with equations taking into account individual characteristics, such as sex, age and Body Mass Index, and not rely on the 1-MET standard.

## 1. Introduction

Resting metabolic rate (RMR) is defined as the energy expended by the body in a resting condition. It accounts for the largest portion of a subject’s total daily energy needs (60%–70%), the remainder being accounted for by diet-induced thermogenesis (~10%) and physical activity-induced energy expenditure (typically ~20%–30%, depending on activity level) [1,2]. RMR can be determined using indirect calorimetry, which allows estimates of energy expenditure to be obtained from measures of carbon dioxide production and oxygen consumption.

Typically, the RMR is measured with a subject physically and mentally at rest, in a supine position, after an overnight fast and in a thermo-neutral environment, to prevent activation of non-RMR heat-generating processes [2]. Because of the relatively high cost and limited availability of the necessary equipment, the time needed for the measurements, the prerequisite that subjects be in a fasted and rested state, and the need for adequately trained personnel, formulas for estimating RMR are frequently applied in clinical and field settings instead of indirect calorimetry measurements [3].

The term “Metabolic Equivalent” (MET) is commonly used by exercise physiologists, nutritionists and the medical community, in circumstances when the RMR is not directly measured. It is defined as the quantity of oxygen consumed by the body from inspired air under resting conditions, that is, 3.5 mL of oxygen per kg body mass per minute (3.5 mL O_2_/kg × min) or ~1 kcal per kg body mass per hour (1 kcal/kg × h) [1]. The conventional 1-MET value is mainly used to express the energy cost of physical activities as a multiple of the RMR (Metabolic Equivalent of Task) [1]. Likewise, the MET concept provides a convenient method to describe the functional capacity or exercise tolerance of an individual. For example, maximal oxygen uptake, usually normalized for body mass, may be translated into METs by dividing it by 3.5, thus providing a unitless and convenient means of referring to a subject’s exercise capacity independent of stature or body composition [4]. The MET concept is also useful in defining a repertoire of physical activities in which a person may participate safely, without exceeding a prescribed intensity level [5].

However, the widespread application of the conventional 1-MET value has begun to be questioned, because it does not apply well to all individuals, nor to all population subgroups [6]. This is not surprising, given that the value was derived from measurements of the resting oxygen consumption of one person, a 70-kg, 40-year-old male, while sitting quietly in a chair [1]. Recently, McMurray et al. examined studies published between 1980 and 2011 on the RMR of adults and analysed the effects of common demographic (sex and age) and anthropometric (body mass index (BMI)) characteristics on RMR variability [7]. They found that the RMR (expressed in kcal/kg × h) is higher in men compared with women, lower in older than in younger adults, and lower in overweight than in normal weight subjects. It was concluded that no single value for RMR is appropriate for all adults, which questions the longstanding adherence to and the indiscriminate use of the conventional 1-MET value.

Despite those reported effects of demographic and anthropometric characteristics on RMR, there is still a paucity of data comparing RMR of other specific population subgroups with the conventional 1-MET value, particularly in youths, pregnant and non-pregnant women, or physically active subjects. These population sub-groups are particularly important, because other factors like growth (adolescence), foetal demands (pregnancy) and energy costs of milk synthesis (lactation) could significantly influence RMR and, thus, cause its value to deviate from the conventional 1-MET value. In addition, there are at present no data examining the RMR of these specific subgroups while taking into consideration their physical activity levels (PAL), that is, their total energy expenditure in multiples of RMR. The purpose of this study is to examine the RMR of adolescents, pregnant and non-pregnant women, as well as active male subjects, using RMR data acquired during several metabolic studies performed in our laboratories, and to compare the measured RMR values with the conventional 1-MET values.

## 2. Materials and Methods

### 2.1. Participants

In the course of several research activities recently performed by our research groups, RMR and PAL were measured in 100 adolescent males and females (unpublished data), 46 pregnant women (35–41 weeks of gestation (mean of 38 weeks)), a subgroup of the same women (*n* = 27) after their pregnancy (24–53 weeks postpartum (mean of 40 weeks)) [8], and 30 active adult males (physical activity level (PAL) of 1.9) [9]. Those data are assembled here for the purpose of comparing the RMR of these specific population sub-groups with the conventional 1-MET value. All study participants were Caucasian healthy subjects. Adolescent and active male subject data were collected at the Swiss Olympic Medical Centre, Magglingen, Switzerland, and data from the pregnant and post-pregnant women were collected at the maternity unit of the University Hospitals of Geneva, Switzerland. All subjects gave their written informed consent to participate. The ethical committees of the respective study sites (Kantonale Ethikkomission Bern (KEK), Bern, No. 230/12 (22 February 2013), No. 086/12 (26 June 2012); Commission centrale d’ethique de la recherche sur l’être humain*,* Genève, No. 09/031 (26 June 2009)) approved the original studies. Subject characteristics are presented in Table 1.

### 2.2. Anthropometric Data

Body weight was measured to the nearest 0.01 kg using a calibrated beam scale (Seca Ltd., Hamburg, Germany) and body height to the nearest 0.01 cm using a height rod (Seca Ltd.), with subjects in underwear and without shoes. BMI was calculated for all subgroups and classified into under-, normal-, overweight and obese using the following categories: For active adult males and adult non-pregnant females according to standard classification [10]; for pregnant females according to Institute of Medicine (IOM) categories for pregnant women [11]; and for adolescents by use of age- and sex-specific percentiles [12,13]. In pregnant women, gestational age was assessed based on the last menstrual period, or based on a first trimester ultrasound measurement if a difference of 1 week between the two estimates was detected. Pubertal development (Tanner stage) of the adolescents was assessed from secondary sexual characteristics (breast development and pubic hair) in girls, and genital development (of testes and penis) and pubic hair in boys [14,15]. The reliability and validity of the scale has been published elsewhere [16].

### 2.3. Resting Energy Expenditure

RMR was assessed by indirect calorimetry using ventilated hood systems; either a Moxus Metabolic System (AEI Technologies Inc., Bastrop, TX, USA) in the adolescent and active male adults, or a Deltatrac II metabolic monitor (Datex-Ohmeda, Helsinki, Finland) in the pregnant women, under the standardized conditions described below. Reliability and validity of the devices have been reported elsewhere [17,18].

In short, calibration of the gas analysers and the flow measurement module was carried out before each measurement according to the manufacturer’s instructions. The subjects were instructed to arrive in the research unit in the morning, after an overnight fast (12 h), avoiding any strenuous physical effort for a minimum of 24 h before the RMR measurement. After acclimating and relaxing on a bed for 30 min, a ventilated hood was placed over their heads and the measurements started. Oxygen consumption (V’O_2_) and carbon dioxide production (V’CO_2_) were measured for 30 min with the subjects in a supine position and completely at rest in a quiet and thermo-neutral environment (20–22 °C). The first 5 min of data were eliminated as an acclimation artefact. From the remaining 25 min, segments of a minimum of 10 consecutive 1-min measures with <10% coefficient of variation in V’O_2_ and V’CO_2_ were considered as steady-state. Average V’O_2_ and V’CO_2_ values were then used to calculate RMR using the abbreviated Weir equation [19].

### 2.4. Total and Activity-Related Energy Expenditure

Activity energy expenditure (AEE) was estimated by analysing 24-h recordings of heart rate and movement (acceleration). A lightweight (10 g), waterproof combined heart rate and movement sensor (Actiheart; Cambridge Neurotechnology Ltd., Papworth, UK) was clipped onto two standard electrocardiography electrodes (Red Dot Electrode 3560; 3M, Diegem, Belgium) on the left thorax just below the level of the apex of the sternum [20]. The device was worn on the chest day and night for a minimum of 5 and up to 7 consecutive full days with a 15-s data epoch setting. The device was calibrated for each subject using a standard step test. The reliability and validity of the device have been described elsewhere [21,22]. The Actiheart was proven to give accurate estimates of AEE compared with indirect calorimetry during a wide range of activities (from low-through moderate- and high-intensity activities) in children, men, pregnant and post-pregnant women in both laboratory [23,24,25] and field settings [26,27].

### 2.5. Statistical Analyses

Descriptive data and continuous variables are reported as mean ± standard deviation (SD) or 95% Confidence Intervals (CI). All RMR data are expressed in kcal/kg × h and compared with the 1-MET standard value of 1 kcal/kg × h. The data were normally distributed apart from age, height, weight, and BMI, according to the Kolmogorov-Smirnov test. *T*-tests and ANOVA with Scheffé post hoc tests for in between-group comparisons of RMR were also performed. An analysis of covariance (ANCOVA) was additionally used to control for the covariates, such as puberty (Tanner stage) and PAL. Spearman correlation was used to assess the relationship between age, BMI, the RMR, and the time of measurement (pregnancy and postpartum week). *p*-values < 0.05 were considered statistically significant. SPSS software (IBM Corp., Chicago, IL, USA, v.22) was used for data description and statistical analyses.

## 3. Results

**Figure 1:**
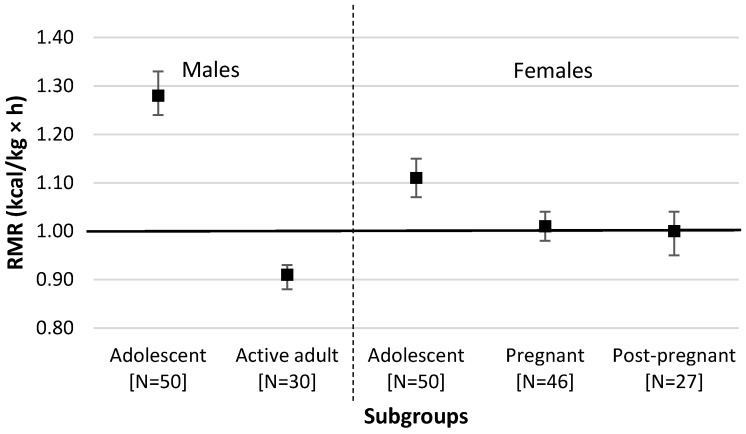
RMR (kcal/kg × h) of various subgroups of the general population. Data are shown as Mean and 95% CI. Solid line = 1.0 kcal/kg × h (conventional 1-MET value).

The overall mean value for RMR from all 203 measurements was 1.08 kcal/kg × h (95% CI = 1.06–1.11), and was significantly different from the conventional 1-MET value (1 kcal/kg × h) (*p* = 0.0001). The RMR of the adolescent males was 1.28 kcal/kg × h (95% CI = 1.24–1.33), and that of the adolescent females was 1.11 kcal/kg × h (95% CI = 1.07–1.14) (Figure 1). The overall adolescent RMR (males and females) differed by Tanner stage 1 (breasts, genitals) (*p* = 0.002) and Tanner stage 2 (pubic hair) (*p* = 0.0001). The RMR of male adolescents did not differ by Tanner stage 1 (*p* = 0.96) but differed by Tanner stage 2 (*p* = 0.006). The RMR of female adolescents differed by Tanner stage 1 and 2 (*p* = 0.042 and *p* = 0.007, respectively).

The RMR of the adolescent males was significantly higher than that of the adolescent females, with or without the effects of puberty stage (Tanner stage 1, Tanner stage 2, or both combined) and PAL (all *p* < 0.0001). The RMR of adolescent males and females were significantly different by age (*p* = 0.002). When analysed separately, the RMR of male adolescents were significantly different by age (*p* = 0.002), while those of the female adolescents were not (*p* = 0.063). The RMR of adolescent males and females were significantly higher than the RMR of any other adult subgroup (all *p* < 0.01, respectively), and the conventional 1-MET value (*p* < 0.0001, respectively).

RMR of adolescent females aged 12–14 years was similar to that of adolescent females aged 15–17 years (1.10 kcal/kg × h (95% CI = 1.03–1.18) vs. 1.11 kcal/kg × h (95% CI = 1.07–1.16), *p* = 1.000). RMR of adolescent males aged 12–14 years old was similar to that of adolescent males aged 15–17 years (1.31 kcal/kg × h (95% CI = 1.24–1.39) vs. 1.26 kcal/kg × h (95% CI = 1.21–1.31), *p* = 0.978). Adolescent females aged 12–14 years had a significantly lower RMR than their male counterparts (*p* = 0.001). Adolescent females aged 15–17 years old had a significantly lower RMR than their male counterparts (*p* = 0.007). RMR of adolescent females aged 12–14 years and of those aged 15–17 years were significantly higher than the conventional 1-MET value of 1 kcal/kg × h (*p* = 0.007 and *p* = 0.0001, respectively). RMR of all male adolescent age subgroups were significantly higher than the conventional 1-MET value (all *p* = 0.0001). There was a tendency for a negative correlation between age and RMR for all adolescents (*r* = −0.2, *p* = 0.06).

**Figure 2:**
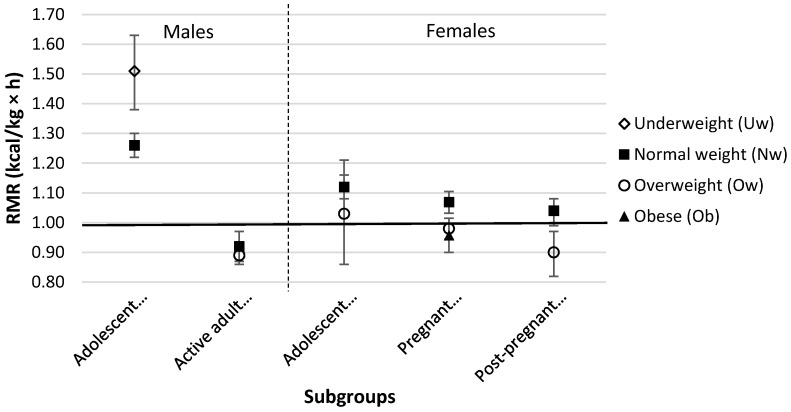
RMR (kcal/kg × h) for Body Mass Index (BMI) (kg/m^2^) subgroups of the general population [10]. Classification of BMI for pregnant females and adolescents is shown according to IOM categories [11] and age- and sex-specific percentiles [12,13], respectively. Data are shown as Mean and 95% CI. Solid line = 1.0 kcal/kg × h (conventional 1-MET value). Uw = underweight, Nw = normal weight, Ow = overweight, Ob = obese.

The underweight male adolescents (*n* = 5) had significantly higher RMR compared with normal weight male adolescents (*n* = 44) (1.51 kcal/kg × h (95% CI = 1.39–1.63) vs. 1.26 kcal/kg × h (95% CI = 1.22–1.30), *p* = 0.001) (Figure 2). The RMR of both underweight and normal weight male adolescents were significantly higher than the conventional 1-MET value (*p* < 0.0001). RMR of the normal weight and overweight female adolescents were similar (1.12 kcal/kg × h (95% CI = 1.08–1.16) vs. 1.03 kcal/kg × h (95% CI = 0.86–1.21), *p* = 0.06). The RMR of the normal weight female adolescents were found to be significantly higher (*p* < 0.0001) than, and those of the overweight adolescents similar (*p* = 0.63) to, the conventional 1-MET value.

The RMR of the pregnant female subjects was not different from that of the post-pregnant female subjects (1.01 kcal/kg × h (95% CI = 0.98–1.04) vs. 1.00 kcal/kg × h (95% CI = 0.95–1.04), *p* = 0.997) (Figure 1). The RMR of pregnant and post-pregnant women were not influenced by the time of measurement (gestational and postpartum week) (*r* = −0.06, *p* = 0.7 and *r* = 0.13, *p* = 0.5, respectively). The RMR of pregnant and post-pregnant women were not significantly different from the conventional 1-MET value (*p* = 0.58 and *p* = 0.86, respectively). Thirteen of the post-pregnant female subjects were lactating, while 14 were not, but their RMR/kg × h was not influenced by lactation status (1.01 kcal/kg × h (95% CI = 0.95–1.07) vs. 0.98 kcal/kg × h (95% CI = 0.92–1.05), *p* = 0.54).

The PAL of 1.9 in active male subjects was significantly higher that the PAL of 1.7 in post-pregnant female subjects (*p* = 0.001). No significant difference in RMR was found between adult male and post-pregnant female subjects (0.91 kcal/kg × h (95% CI = 0.84–0.93) vs. 1.00 kcal/kg × h (95% CI = 0.95–1.04), *p* = 0.09)), including when adjusted for PAL (*p* = 0.26). The RMR of the pregnant female subjects was significantly higher than those of the active male subjects (1.01 kcal/kg × h (95% CI = 0.98–1.04) vs. 0.91 kcal/kg × h (95% CI = 0.88–0.93), *p* = 0.01). Active adult male subjects had significantly lower RMR than the conventional 1-MET value (*p* = 0.001). A negative correlation was found between age and RMR for active adult males (*r* = −0.78, *p* < 0.001), females excluding pregnant women (*r* = −0.33, *p* = 0.003), and females including pregnant women (*r* = −0.36, *p* < 0.01).

Figure 2 shows the adult RMR data for the different BMI groups. The RMR of normal weight and overweight active adult males were similar (0.92 kcal/kg × h (95% CI = 0.87–0.97) vs. 0.89 kcal/kg × h (95% CI = 0.87–0.97), *p* = 0.322). The RMR of normal weight and overweight active adult males were significantly lower than the conventional 1-MET value of 1 kcal/kg × h (*p* = 0.006 and *p* = 0.0001, respectively). The RMR of normal weight post-pregnant females was significantly higher than that of the overweight post-pregnant females (1.03 kcal/kg × h (95% CI = 0.99–1.08) vs. 0.88 kcal/kg × h (95% CI = 0.82–0.97), *p* = 0.009). While the RMR of normal weight post-pregnant women was not significantly different from the conventional 1-MET value (*p* = 0.19), that of the overweight post-pregnant women was significantly lower (*p* = 0.007).

The RMR of normal weight, overweight, and obese pregnant females were not significantly different from the conventional 1-MET value (all *p* > 0.05). There was a significant negative correlation between BMI and RMR for post-pregnant females (*r* = −0.54, *p* = 0.004), adult females (including pregnant females) (*r* = −0.38, *p* = 0.001), and active adult males (*r* = −0.37, *p* = 0.045).

## 4. Discussion

Previous studies conducted with adults have shown that indiscriminate use of the standard MET (1 kcal/kg × h) leads to considerably greater error than using actual RMR measurements [6,7]. We have now extended this observation to other populations including adolescents (aged 12–17 years), pregnant women (35–41 weeks of gestation), post-pregnant women (24–53 weeks postpartum) and active men (PAL = 1.9). Our results illustrate the considerable potential for error when using conventional 1-MET value in adolescents and adult physically active men, and to a lesser extent, in women during and after pregnancy. It follows that, depending on an individual’s characteristics, METs should be used with caution.

When expressed in kcal/kg × h, the mean RMR of both adolescent males and females were significantly higher than the mean RMR of any other adult subgroup and the conventional 1-MET value. In teenagers (age group 11 to 17 years), the values in males were significantly higher than those in females, regardless of obesity status, puberty stage or age.

Holliday et al. studied the relationship between metabolic rate and body mass in different age groups and reported that RMR/kg in humans during growth could be as high as 56 kcal in infants below 6 months, weighing about 6 kg (2.3 kcal/kg × h) [28]. The rates of increase of RMR and body mass are comparable and constant up to 10–12 kg and are independent of sex. RMR then progressively decreases with age and reaches 25–30 kcal/kg by age 20 (1.04–1.25 kcal/kg × h) [29], with a modest surge during puberty. Beyond that, the relative rate of increase in RMR is much slower than that of body mass, with a second, less obvious change, occurring at 30–38 kg body weight [29]. This progressive decrease in RMR/kg × h with age is a result of the relatively slower growth of the highly active organs (brain, heart, kidney, livers and lung), compared with muscle and/or total body mass [29].

According to the Food and Agriculture Organization of the United Nations/World Health Organisation/United Nations University (FAO/WHO/UNU) Human Energy Requirements report, the energy needed during resting state is 33 kcal/kg to 27 kcal/kg for boys aged 12 to 17 years (mean = 30 kcal/kg (1.3 kcal/kg × h)), and 29 kcal/kg to 25 kcal/kg for girls of the same age group (mean = 27 kcal/kg (1.1 kcal/kg × h)) [30]. These data were estimated using the Schofield equation [31]. We reviewed data on healthy, normal-weight adolescents whose RMR was measured using indirect calorimetry and found similar results [30,32,33,34,35,36,37,38,39,40,41,42,43,44,45], with girls (*n* = 512) expending, on average, 1.17 kcal/kg × h, and boys (*n* = 480) 1.32 kcal/kg × h. Finally, our own measurements presented in this study yielded similar results (Table 1), showing that the RMR of adolescents is significantly higher than the conventional 1-MET value, with BMI status and sex influencing the results, regardless of puberty stage.

The RMR values of the pregnant women we assessed did not differ significantly from the conventional 1-MET value, regardless of their obesity status. Pregnancy was shown to increase absolute RMR in concert with body weight gain, in about the same proportion [46]. The most significant alteration occurs in the last trimester when the RMR increases by the equivalent of an extra 200–400 kcal/day (~20%) [8,47,48,49,50,51,52]. This extra energy requirement nevertheless varies considerably between women, and the factors responsible for this variability are largely unknown [50,51,52,53]. Weight gain during pregnancy comprises the products of conception (foetus, placenta, and amniotic fluid) as well as the increases in several maternal tissues (uterus, breasts, blood, fat stores, and extracellular as well as extravascular fluid) [54]. Fat deposition takes place predominantly in maternal adipose tissue (~85%), the rest being stored in the foetus (~15%) [52,55]. The increase in body fat, considered to be a metabolically less active tissue than fat-free mass, could be expected to decrease the RMR relative to body weight (RMR/kg), while the presence of the metabolically active foetus could be expected to have the opposite effect. Indeed, our data indicate that the RMR/kg was unchanged in women who are in the third trimester of pregnancy vs. post-pregnant women, even when the increase in body weight during pregnancy was around 15 kg (Table 1). Furthermore, the RMR of both the post-pregnant (normal and overweight) and pregnant women were similar to the conventional 1-MET value. The overweight post-pregnant women studied separately had a significantly lower RMR than the conventional 1-MET value, presumably resulting from a decrease in the proportion of fat-free mass.

It should also be noted that the RMR of the post-pregnant female subjects was found to be independent of lactation status. Our results are in line with other longitudinal studies that found no difference in RMR between lactating and non-lactating women [56], even when adjusting by differences in metabolically active muscle mass [57,58]. Some studies, though, showed a modest increase in RMR in lactating women compared with their pre-pregnant RMR, but these results were presented in absolute values, without taking into consideration the differences in body composition nor the higher residual body weight in the lactating women [59,60].

The RMR of lactating women would be expected to be elevated as a result of the energy cost of milk synthesis [61]. The fact that RMR did not increase during lactation in our study might therefore indicate that one or more components of the mother’s own metabolism must have been suppressed, suggesting evidence for some limited energy-sparing adaptations.

We found a negative correlation between age and RMR for all adult subgroups (active male, post-pregnant and pregnant women). The decline in RMR was reported to be mainly attributable to the progressive absolute and proportional loss of fat-free mass observed with aging [62], and proportional changes in its metabolically active components [63,64]. However, a decline in RMR with age, independent of any age-related changes in fat free mass, was observed in some studies to result from other factors, such as cell loss of organs and tissues, expansion of the extracellular compartments, lower RMR per unit cell mass [65] and organ weight changes [66]. All of these factors appear to have an impact on RMR.

The RMR of the adult active males were similar to those of the non-pregnant female adults, but in the former, it was lower than the conventional 1-MET value, regardless of BMI. This observation was previously reported by McMurray et al. [7], who reviewed RMR data in 113 adult men (~0.85 kcal/kg × h). Our measurements of RMR in men yielded slightly higher values (0.89 kcal/kg × h). The RMR of male subjects would have been expected to be higher than those of the non-pregnant female subjects as the muscle mass, the metabolically active tissue, is usually higher in males compared with females. Certain studies support a lower RMR in women than in men even independently of differences in body composition [67,68]. The lack of body composition data prevented us from further examining the potential influences of metabolically active muscle mass on the measured RMR.

The surprisingly similar RMR values per kg in men and women, which are not often reported, can partly be explained by the physical characteristics of the two groups: The absolute difference between males and females was 19 kg in weight (mean of 81 kg in males vs. 62 kg in females), 14 cm in height and 3 units BMI greater in males, although both males and females had BMI within the reference range. We know that there is a negative curvilinear relationship between body weight and RMR per unit body weight in both males and females, with a downward shift in females; this suggests that the large gap in body weight in males as compared with females would have attenuated the relative difference in RMR between females and males.

Furthermore, it should be noted that the RMR of the adult men presented in this study were measured after an 8-week-long exercise intervention program (daily activities for ~90 min at moderate and ~10 min at high intensity levels (PAL = 1.9)) leading to a loss of body mass. The PAL of the active male subjects were significantly higher than the PAL of the non-pregnant women. The engagement in such high activity levels in combination with a negative energy balance might have decreased the RMR of these active male subjects [69].

Some cross-sectional [70,71] and longitudinal [72,73] studies reported no changes in RMR with endurance or resistance training in the absence of dietary restriction. Other cross-sectional [70,74], and longitudinal studies [75] suggest that exercise training without dietary restriction increases RMR, but the alterations are thought to represent an acute effect of exercise (excess post-exercise oxygen consumption (EPOC)), rather than a long-term adaptation to aerobic training [76,77]. One possible explanation for these discrepant results could be the timing of the RMR measurement in relation to the time of the last exercise bout. The latest reviews on best practices for performing RMR measurements in healthy individuals indicate that the duration of the effects of various types of exercise on RMR is unknown, but suggest “refraining from physical activity before the RMR measurement for a period of time (for example, 12–48 h for moderate to vigorous physical activity)” [78]. The subjects in our studies performed mainly physical activity of moderate intensity, but were instructed to refrain from any activity for a minimum of 24 h before the RMR measurement, which goes in line with the current recommendations.

Extreme exercise interventions, however, may induce reductions in RMR, in spite of the increases in lean tissue mass that they cause [69]. Some authors have proposed that non-physical activity energy expenditure adapts dynamically to variations in physical activity in order to maintain total energy expenditure within some narrow physiological range, thus decreasing the RMR with the increase in exercise-induced energy expenditure [79].

Figure 2 shows the influence of obesity status on RMR. It should be emphasized that “metabolic unit” uses a per weight ratio model (kcal/kg × h), which introduces an additional error in subjects of different body sizes. For example, an obese person having an identical amount of metabolically-active muscle mass as compared to a normal person, would have a lower 1-MET value simply because the RMR in the numerator would be divided by a total body mass in the denominator that is much higher in the obese than in the normal weight subject.

The use of the per-ratio model facilitates comparison between individuals, assuming that: (1) the RMR rises proportionately to the increase in body mass so that the value per kg remains constant; and that (2) the relationship between the measurements is linear and passes through the origin [80]. However, the relationship between RMR and body mass was shown to have, in fact, a non-zero intercept [81]. The traditional ratio approach is therefore precluded for data comparison and it was suggested that additive adjustments based on the regression line (non-zero intercepts) be used instead [82,83]. Nevertheless, the regression-based approach still assumes a linear relationship between the variables, even though such variables are known to have a curvilinear relationship. Consequently, a curvilinear “allometric or power function model” was recommended for use since it was theoretically, physiologically and mathematically superior to the linear models [84].

A limitation of this study is that we did not perform body composition assessment within the scope of our several metabolic research activities in adolescents, pregnant, non-pregnant women and active male subjects whose measured RMR data are assembled herein. Body composition data would have allowed us to further examine the factors potentially influencing the discrepancies between the measured RMR and conventional 1-MET value. Nonetheless, we succeeded in reporting the deviations of measured RMR from the conventional 1-MET as expressed in terms of body mass ratio (1 kcal/kg × h), which was the main objective of the study.

The strength of our study is that it is, to our knowledge, the first investigation to compare measured RMR with the commonly-accepted 1-MET value in a population comprising adolescents of both sex, active adults, and both pregnant and post-pregnant women. McMurray et al. [7] and Byrne et al. [6] examined the effects of age, sex and obesity status on RMR, but that study focused only on adults. In addition, they admitted that they could not guarantee that data from marginal studies did not affect their findings, because many studies did not report the duration of postprandial state. Our data were obtained in our laboratories by adequately trained personnel, under strictly-defined conditions regarding the time needed for the measurements, and the prerequisite that subjects be in a fasted state. In addition, we had available data on activity-related energy expenditure for our participants, which allowed us to examine its potential influences. The differences between the measured and the conventional 1-MET value in adolescent, pregnant and active adult populations and the potential influence of sex, age, obesity status and physical activity level on RMR are valuable findings of great importance for public health purposes. It is particularly important to study and compare the measured RMR of these population sub-groups to the conventional 1-MET value, because the RMR values of these groups are influenced not only by sex, age, obesity status or physical activity level, but also by other factors, such as growth (adolescence), foetal demands (pregnancy), and energy cost of milk synthesis (lactation). To our knowledge, our study is the first to provide evidence that the 1-MET value applies to both, and does differ in, pregnant and non-pregnant women.

## 5. Conclusions

We report discrepancies between measured RMR and the 1-MET standard in adolescents (aged 12–17 years), pregnant women (35–41 weeks of gestation), post-pregnant women (24–53 weeks postpartum) and active men (PAL = 1.9), extending earlier observations in adults. It follows that indiscriminate use of the conventional 1-MET value is likely to bias the true relative energy cost of exercise expressed in MET, and may have implications for the formulation of exercise prescription for specific population subgroups. We recommend that instead of using the 1-MET standard, the RMR is estimated using validated equations taking into account at least sex, age and BMI.

## Figures and Tables

**Table 1:** Characteristics of the subjects.

Subjects	Males		Females		
	Adolescent (*n* = 50)	Active Adult (*n* = 30)	Adolescent (*n* = 50)	Pregnant (*n* = 46)	Post-Pregnant (*n* = 27)
Age (years)	14.7 ± 1.7 ^#^	29.7 ± 7.2	14.8 ± 1.6 ^#^	31.3 ± 5.5	31.8 ± 5.3
Height (cm)	169 ± 11	180 ± 6.9 *	164 ± 6 °	166 ± 6	166 ± 7
Weight (kg)	56.4 ± 11.3 ^§,&^	81.0 ± 7.3	57.3 ± 8.4 ^§,&^	77.2 ± 12.6	62.3 ± 10.6 ^§,&^
BMI (kg/m^2^)	19.6 ± 2.3 ^#^	25.5 ± 2.3	21.2 ± 2.7 ^§,&^	28.1 ± 4.1	22.5 ± 3.0 ^§,&^
PAL	1.6 ± 0.2	1.9 ± 0.2 *	1.6 ± 0.1	1.5 ± 0.2 °	1.7 ± 0.3 ^§^

Data are shown as Mean ± SD. BMI = body mass index; PAL = physical activity level; * significantly different from all other subgroups (*p* < 0.05); ^&^ significantly different from male adults (*p* < 0.05); ^§^ significantly different from pregnant (*p* < 0.01); ° significantly different from male adolescents (*p* < 0.05); ^#^ significantly different from male adults, female adults, and pregnant (*p* < 0.05).

## Abbreviations

The following abbreviations are used in this manuscript:

MET	Metabolic equivalent
RMR	Resting metabolic rate
PAL	Physical activity level
TEE	Total energy expenditure
BMI	Body mass index
V’O_2_	Oxygen consumption
V’CO_2_	Carbon dioxide production
AEE	Activity energy expenditure
DLW	Doubly labelled water
CI	Confidence Intervals
